# The orientation of homing pigeons (*Columba livia* f.d.) with and without navigational experience in a two-dimensional environment

**DOI:** 10.1371/journal.pone.0188483

**Published:** 2017-11-27

**Authors:** Julia Mehlhorn, Gerd Rehkaemper

**Affiliations:** Research Group “Comparative Neurobiology and Evolutionary Research”, Institute of Anatomy I, University of Düsseldorf, Düsseldorf, Germany; University of New England, Australia, AUSTRALIA

## Abstract

Homing pigeons are known for their excellent homing ability, and their brains seem to be functionally adapted to homing. It is known that pigeons with navigational experience show a larger hippocampus and also a more lateralised brain than pigeons without navigational experience. So we hypothesized that experience may have an influence also on orientation ability. We examined two groups of pigeons (11 with navigational experience and 17 without) in a standard operant chamber with a touch screen monitor showing a 2-D schematic of a rectangular environment (as “geometric” information) and one uniquely shaped and colored feature in each corner (as “landmark” information). Pigeons were trained first for pecking on one of these features and then we examined their ability to encode geometric and landmark information in four tests by modifying the rectangular environment. All tests were done under binocular and monocular viewing to test hemispheric dominance. The number of pecks was counted for analysis. Results show that generally both groups orientate on the basis of landmarks and the geometry of environment, but landmark information was preferred. Pigeons with navigational experience did not perform better on the tests but showed a better conjunction of the different kinds of information. Significant differences between monocular and binocular viewing were detected particularly in pigeons without navigational experience on two tests with reduced information. Our data suggest that the conjunction of geometric and landmark information might be integrated after processing separately in each hemisphere and that this process is influenced by experience.

## Introduction

A large amount of research has been conducted to understand which mechanisms and environmental properties enable animals to navigate accurately [1,2]. Many studies have shown that animals can encode and use multiple sources of information to locate a goal. For navigation within a familiar environment e.g., visual landmarks are an important source of information and are used by many species, including birds, [3–6]. Additionally, several studies have revealed that the geometry as defined by continuous surfaces can also deliver an important cue for an animal to obtain a sense of bearing for orientation [3,7,8,9,10]. However, the extent to which landmark and geometric information are relied upon and how much of the available information is actually used seems to differ across species and with experience [7,11,12].

Homing pigeons are an excellent model for investigating spatial cognition and/or orientation mechanisms. They use several orientation mechanisms (e.g. sun compass, magnetic compass, olfactory cues), but there is a general agreement that pigeons rely at least on visual cues or landmarks for flights within their familiar home ranges (<5 km) or for finding their home loft from familiar nearby releasing sites [2,13–17]. Kelly et al. [4] have already shown that Silver King pigeons are able to encode landmark and geometric information in an experimental environment (rectangular enclosure with three-dimensional objects or two-dimensional panels in each corner). However, the extent to which the pigeons later relied on geometric and landmark information is influenced by the bird’s initial experience in the experimental situation, e.g. whether they were trained with or without landmark information before being tested with landmark and/or geometric information. Most of these studies have used a navigable open-field task, a kind of “spatial arena” in the form of a rectangular enclosed container. That pigeons are able to re-orientate, at least under certain condition, according to configural geometry in a rectangular array of landmarks was also shown by Pecchia et al. [18]. The role of landmarks and geometry in pigeon spatial cognition has also been investigated by using two-dimensional touch-screens [19]. It is quite apparent that encoding information within a three-dimensional (3-D) rectangular room could differ from encoding information within a two-dimensional (2-D) touch-screen task, because food is not found at the goal but instead dropped in a feeder in a different location and the search space is vertical and viewed at close range. Thus, the touch-screen task presents stimuli mainly to the short-distance binocular frontal field of the pigeon whereas in a 3-D rectangular room (or in natural situations) also the long-range monocular fields are called into play [20,21]. Kelly & Spetch [19] also used a touch-screen task conducted within a standard operant chamber. They trained pigeons to search for a hidden target area in images showing a schematic rectangular environment (served as geometric information). Various graphic stimuli in the corners of the rectangle served as landmarks. They revealed that pigeons are able to use the rectangular properties to locate the goal and that they could also use a single featural cue at an incorrect corner to distinguish between the correct corner and the geometrically equivalent corner.

Previous studies have mostly used Silver King pigeons, which were originally bred for meat production and have a huge habitus and limited flying and orientation capabilities. Thus, these animals are not really appropriate to investigate orientation. In the present study we used for the first time homing pigeons originated from high performance racing breeds and thus, with strong navigational skills. Corresponding to their excellent homing abilities, the brains of homing pigeons are well adapted to homing with e.g. larger hippocampi and olfactory bulbs in comparison to other pigeon breeds or even rock doves, the wild ancestor of homing pigeons [22,23]. In a previous study, we revealed that hippocampal volume depends on navigational experience and associated flying. Pigeons with a lot of navigational experience have a larger hippocampus volume than non-experienced or less experienced pigeons [24]. This was consistent with earlier findings that homing has its structural base in the hippocampus [2,25]. Hippocampus lesion experiments in homing pigeons showed that the hippocampus appears to be essential for the learning of map-like, spatial representations of environmental stimuli used for navigation and complements the navigational map of the homing pigeon. Under laboratory conditions, it can be seen that hippocampus-lesioned pigeons are particularly unable to use geometric information in contrast to feature cues [26].

Furthermore, we previously showed that experience not just has an influence on hippocampus volume, but also on brain lateralization which is reflected in homing pigeons’ brain structure volume [27]. Pigeons with navigational experience show a more lateralized brain than pigeons without navigational experience. The brain structures affected are the hippocampus, the apical hyperpallium, the nidopallium, and the mesopallium. These structures are all involved in processing spatial or visual information [28,29,30]. Functional cerebral lateralization has been well documented in many species including birds [31,32]. Studies have documented right-left differences also in avian visual processing [33,34,35] and aspects of spatial learning and memory [36–41].

In the present study, we investigated how navigationally experienced and non-experienced pigeons encode featural (landmark) and geometric information when presented with a 2-D schematic of a rectangular environment, based on Kelly & Spetch [19]. This study is of interest because it complements previous investigations in pigeons that have examined the use of discrete landmarks for spatial search within 2-D touch-screen environments. But here, we used homing pigeons whose navigational abilities topped all other pigeon breeds respectively such as fancy or non-homing breeds like Silver King pigeons. The homing pigeons of the present study were bred for highly competitive pigeon races with distances up to 800 km and therefore should have strong navigational skills. Using such pigeons is an outstanding feature of this study.

Besides, the pigeons carried out all experiments first with both eyes and then again with monocular occlusion. Diekamp et al. [42] have demonstrated a substantial effect of monocular occlusion on homing performance, which corresponds to anatomical lateralization. In pigeons, visual information crosses completely to the contralateral optic tectum and the contralateral telencephalic hemisphere, and there are no large interhemispheric commissures as seen in mammals [30]. Thus, visual input to the right eye is processed mainly in the left brain hemisphere, and visual signals from the left eye are processed in the right brain hemisphere. Surely, there are as well connections between e.g. the optic tectum and the nucleus rotundus of both hemispheres and this might affect the lateralization of information taken in by the different eyes including behavioural consequences [43,44], but nonetheless, the design of this study should provide insights into the functional lateralization of the brain as well as which hemisphere encodes geometric or featural information.

We anticipated that non-experienced pigeons would show a poorer or at least different performance in comparison to experienced pigeons. Furthermore, we expected that the results would differ depending on binocular or monocular viewing.

## Materials and methods

### Subjects

Twenty-eight homing pigeons (*Columba livia* f.d.) originating from the same breeding stock of our own pigeon breed were raised in the same loft under identical conditions. After fledging, they were randomly assigned to two experimental groups: 17 of them (9 males, 8 females) were allowed to leave the loft to fly around, gained navigational experience, and participated successfully in pigeon races. This meant that they participated in at least 6 (four pigeons) to 10 (seven pigeons) races with distances of 50–284 km. The other six pigeons participated in 7 (three pigeons) or 8 (three pigeons) races. Thus, the whole flight performance of every pigeon varied between 959 km and 1119 km.

The other 11 pigeons (5 males, 6 females) stayed permanently in the loft and did not acquire the navigational experience of the first group. Yet the size of the loft (250 cm X 190 cm X 190 cm) did still allow these pigeons to fly. Thus, the design of our study ensured that the two pigeon groups differed only with respect to free flying and homing experience. All pigeons were at least six months old before the tests began and experimentally naïve with spatial search tasks. The daily requirement of food was estimated and after the start of the experiment, was provided only in the operant chamber. Grit and water were provided ad libitum.

### Apparatus

The experimental apparatus was a custom-built operant chamber of 34 cm x 33 cm x 26 cm (Biobserve; Bonn, Germany). A color touch-screen monitor (screen size: 25.654 cm, Faytech; Göttingen, Germany) was placed inside the box against the front side. The touch-screen monitor was programmed to detect individual responses (pecks). Food rewards were presented using a standard pigeon feeder, located on the front side of the chamber under the monitor. A small lamp illuminated the feeder when a reward was available. The chamber was connected to a personal computer located next to it. This computer controlled all of the experimental contingencies and recorded the responses. The interior of the chamber was visually monitored on an external screen (LCD TV; Lenco; Nettetal, Germany) fed by a camera (CMOS Color Camera; Conrad Electronics; Hirschau, Germany), which was attached above a transparent window in the roof of the chamber.

### Experimental design

#### Preliminary procedure

**Fig 1:**
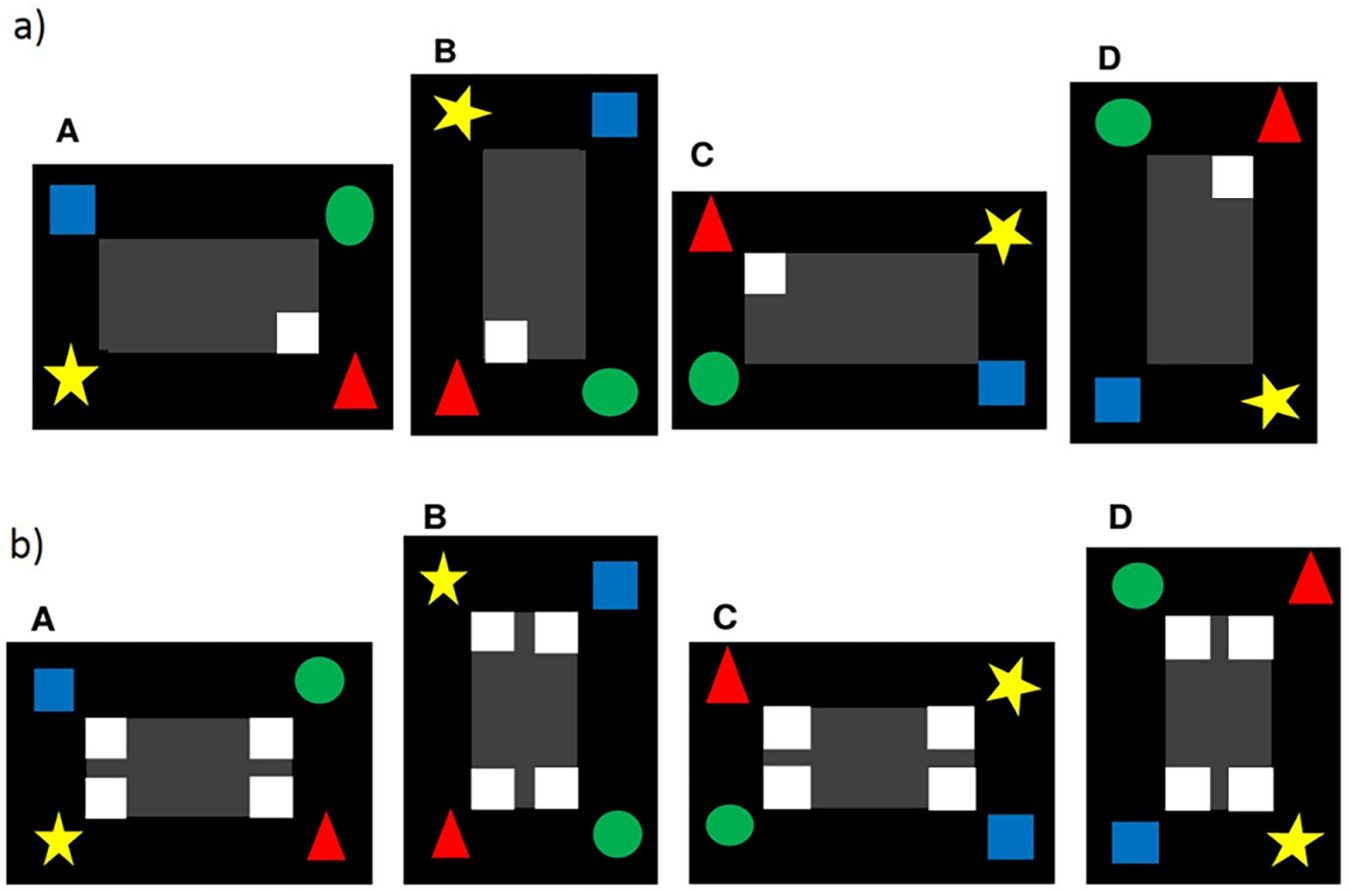
Examples of the stimuli used in *pretraining* (a) and *training* (b). In this example, the red triangle was reinforced. The stimulus rotated clockwise during the experimental session (A: 0°; B: 90°; C: 180°; D: 270°), and the pigeons used both eyes.

All the pigeons received several sessions of training (one session a day per bird) to establish reliable pecking at the monitor. Initially, the pigeons were habituated to the chamber and learned to get food in the chamber by using the pigeon feeder (*shaping*). No stimulus was presented and food was available during the session time which lasted 10 minutes in *shaping*. During the following *autoshaping*, a white square of 30 mm (first step) or 10 mm (second step) was intermittently presented in the middle of the screen against a black background, for 40 trials per session, with a 30 second intertrial intervals. The white square remained on the screen until the pigeon pecked at the square or 10 seconds had elapsed, and then food was presented. If the pigeon pecked the black background during the intertrial interval, the 30 second sequence was interrupted, and the square was presented immediately. After reliable pecking was established (20 or more pecked trials out of the total of 40), the pigeon got food only by pecking the square. Now, the pigeon had to peck the square for a trial to be completed. Again after 20 or more pecked trials, the size of the square was reduced from 30 mm to 10 mm. After the pigeon had completed here at least 20 trials within one session, *autoshaping* was finished and *pretraining* began. If the pigeon did not peck the square for 30 days/30 sessions, it was excluded from experiment. Images for *pretraining* presented a black background with a grey rectangle. Four uniquely colored and shaped landmarks/features (blue square, green circle, yellow star, red triangle) were presented adjacent to the four corners of the rectangle, and each pigeon was randomly assigned one of the four possible corners (associated with one landmark/feature). These four landmarks/features remained visible for all 40 trials per session during *pretraining*. In *pretraining*, the assigned corner of the rectangle contained a white response square (1 cm x 1cm, Fig 1A). Pecks to the white response square were immediately reinforced. *Pretraining* continued until a pigeon pecked the square at least 32 trials (out of the total of 40) on two consecutive sessions. The stimulus rotated clockwise during each experimental session (Fig 1A), and the pigeons were permitted to use both eyes.

In the following *training*, all corners of the rectangle contained a white response square (Fig 1B), but only pecking the correct (assigned) response square was rewarded and completed a trial successfully. *Training* continued until a pigeon completed successfully at least 32 trials (out of the total of 40) on two consecutive sessions. The stimulus rotated clockwise during each experimental session (Fig 1B), and the pigeons were permitted to use both eyes.

Thus, after *training*, all pigeons were conditioned to get food by pecking on one response square which was associated with geometric information from the rectangle (e.g. long side left, short side right of the correct corner/feature) as well as a unique feature or “landmark”.

#### Tests

After *training* all pigeons had to complete four *tests* (one test per day and bird) with again 40 trials total per test. Every *test* consisted of four test cycles, each with six *training* trials followed by four *test* trials. In the *test* trials (as well as in the *training* trials), the pigeons were also rewarded for pecking their assigned corner with respect to their assigned feature/landmark.

To examine whether featural and geometric representations would withstand spatial translations, we carried out as well a *Novel Perspective Test* where the same images were presented as in the training, but now in the lower left or right part of the touch-screen monitor.

Each of the four *tests* was carried out twice, and there were three testing conditions: binocular viewing, left eye occluded, right eye occluded. Thus, each test was done six times in total. For the monocular viewing, all pigeons were fitted with a ring of Velcro strip, attached around both eyes with “Precision Lash Adhesive” (Fing’rs; Stuttgart, Germany). Just before each experimental session, an eyepatch of Velcro strip was attached on this ring and covered the eye completely. This eyepatch was removed immediately after each session. For half of the pigeons, the order of the sessions per test was as follows: binocular viewing, left eye occluded, right eye occluded, binocular viewing, right eye occluded, left eye occluded. For the other half of the pigeons, the order of the sessions per test was: binocular viewing, right eye occluded, left eye occluded, binocular viewing, left eye occluded, right eye occluded. To complete a test session, a pigeon was required to peck during at least 12 test trials, independently from the choice.

The four tests were as follows.

**Fig 2:**
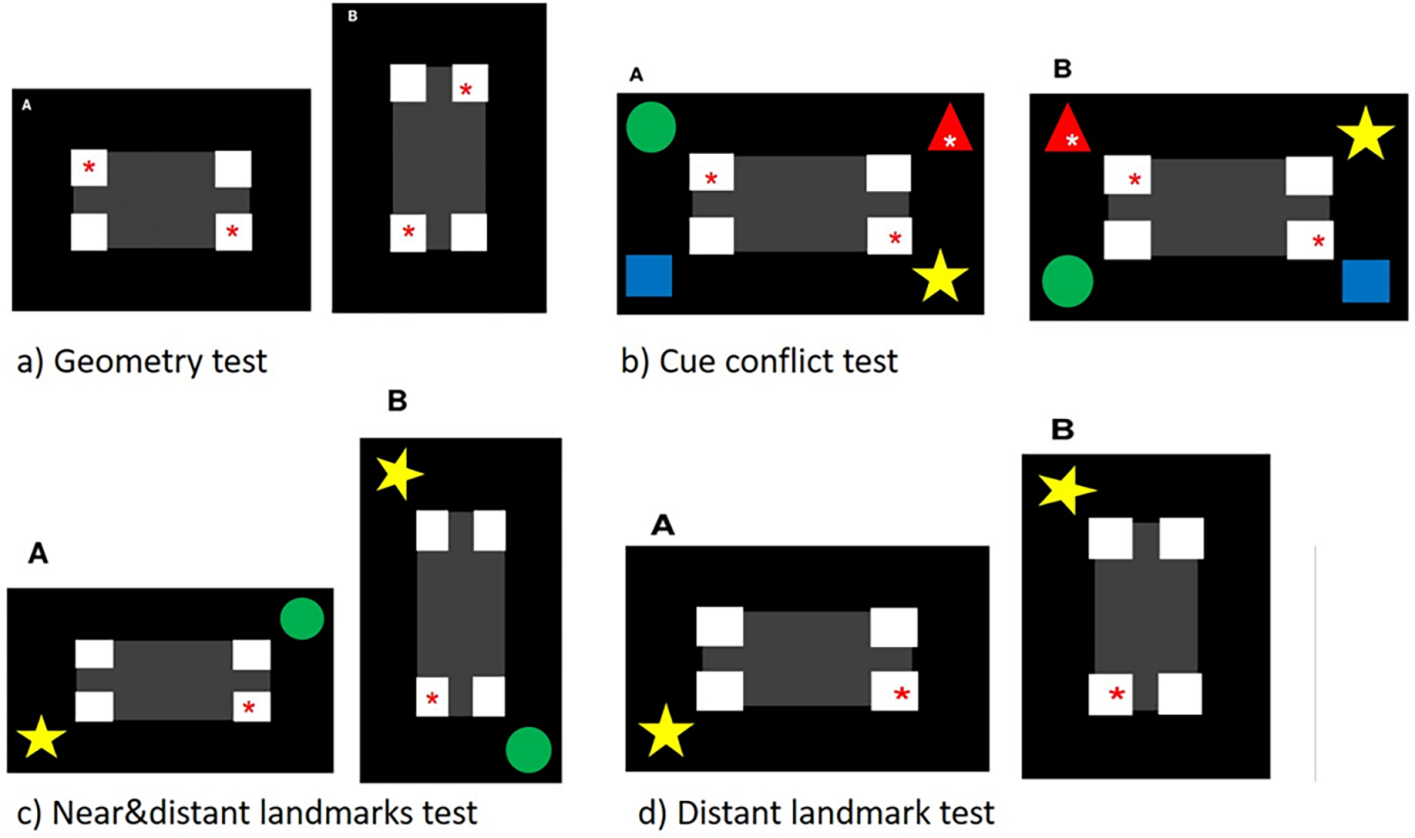
Examples of the stimuli used in the tests. For better demonstration, red (and white) asterisks show the rewarded corner(s)/features (here the red triangle) but were not visible for the pigeon during the test. The stimulus rotated clockwise; two different arrangements are shown (A: 0°; B: 90°).

1. Geometry test (Fig 2A)
  Here, all features/landmarks were removed, so only geometric information was available. Because of this, there were now two correct choices possible, because two corners had the assigned/correct geometric information. Thus, the probability of choosing the correct corner by chance was 50%.
2. Cue Conflict test (Fig 2B)
  In the *cue conflict test*, the features/landmarks rotated counter clockwise around the rectangle. So, there are positions where there was a discrepancy between featural and geometrical information. Here, the correct feature/landmark was not in the corner with the correct geometric information. The pigeons had to decide which kind of information was more important for them for selecting a corner. We laid our focus on the featural information and rewarded the corner with the correct (assigned) feature/landmark. Thus, probability of choosing the correct (landmark) corner by chance was 25%.
3. Near & distant landmarks test (Fig 2C)
  In the *near & distant landmarks test*, the correct feature/landmark and the feature/landmark of the diagonal corner were removed. Thus, both features with the correct geometric information were removed. The other two features/landmarks–the one at the end of the short side of the rectangle (*near* to the correct corner) and the one at the end of the long side of the rectangle (*distant* to the correct corner)–were still shown. The probability of choosing the correct corner by chance was 25%.
4. Distant landmark only test (Fig 2D)
  In the *distant landmark only test*, three features/landmarks were removed: the correct one, the feature/landmark of the diagonal corner and the feature/landmark near to the correct corner (at the end of the short side of the rectangle). The probability of choosing the correct corner by chance was 25%.

Both *landmark tests* were to check whether the pigeons also use features for their choice, which were not rewarded or whether they also learned information about the relative positions of the other landmarks and the goal.

### Data analysis

For analysis, the number of correct pecks was counted and compared in relation to the total number of pecks. We calculated the percentage of choices (“% choice”) made to a particular corner averaged over all pigeons in the particular study group. To rule out the possibility that the pigeons chose the corner/landmark by chance, an ANOVA followed by Fisher’s least significance test (LSD) was first used to compare the test data of both groups to the “by chance” probability of 50% (*geometry test*) or 25% (all other tests). After this, the data were compared statistically by t-test, or ANOVA (on ranks) for comparisons between the groups. Comparisons within the groups were done with parametric and non-parametric tests for dependent data, namely paired t-test, and (Friedman) Repeated Measures Analysis of Variance (on ranks) (FRM ANOVA, RM ANOVA). The level of significance was actual 5%, but for test analyses, we adjusted the alpha level to account for multiple comparisons using the Bonferroni correction; the alpha level was thus set to 0.004 (comparisons between the groups) or to 0.003 (comparisons within the groups). The software package SigmaPlot/SigmaStat version 12.0 was used for all statistical calculations.

All applicable international, national, and/or institutional guidelines for the care and use of animals were followed. The study was approved by the Committee on the Ethics of Animal Experiments of the state of North Rhine-Westphalia (Ref. 84–02.04.2013.A226).

## Results

### Preliminary procedure

**Fig 3:**
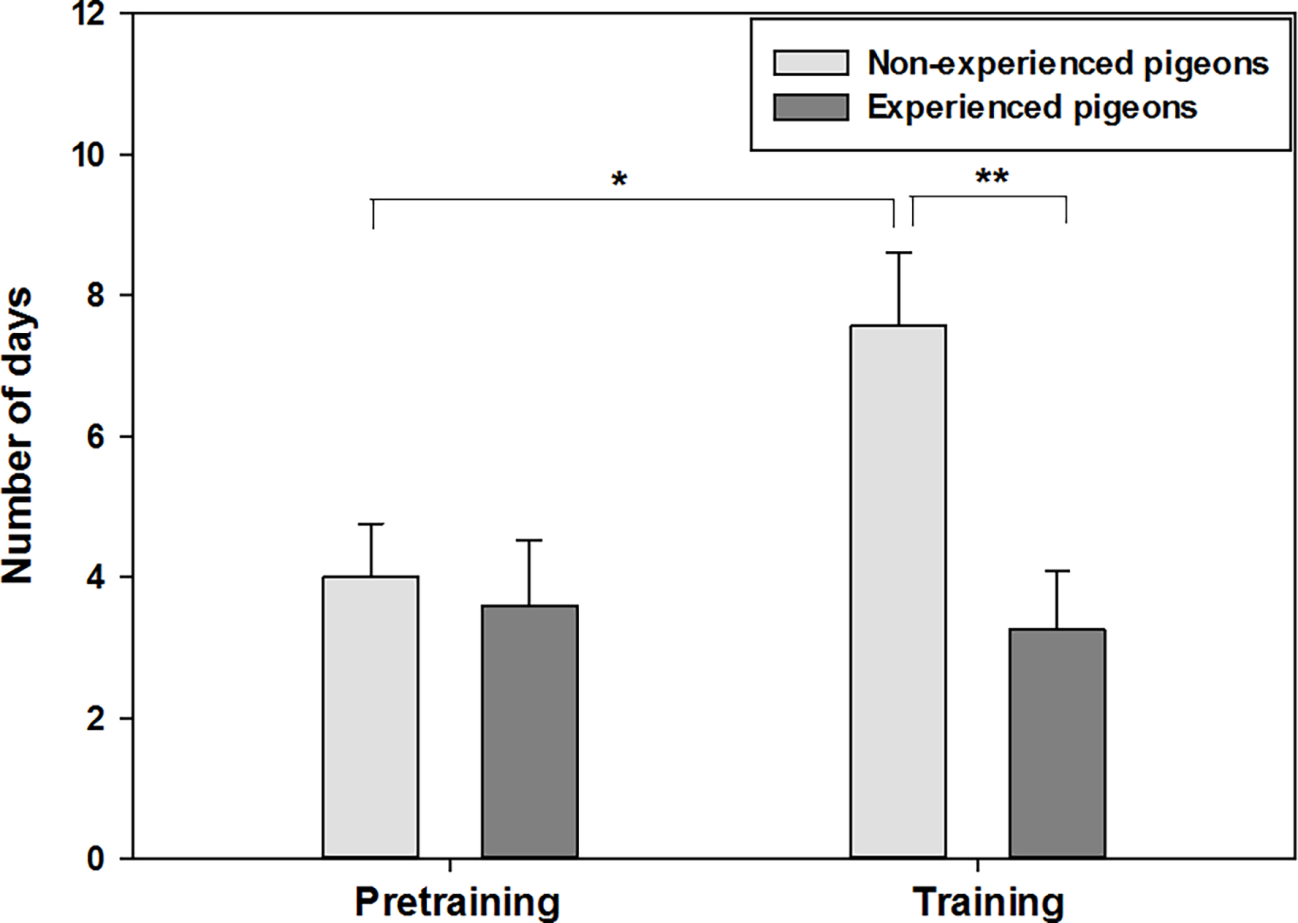
Required days for *pretraining* and *training* of non-experienced (n = 7) and experienced pigeons (n = 10). Error bars represent the standard errors of the mean (* = 0.029, ** = 0.006).

Non-experienced pigeons needed significantly more time than experienced pigeons to habituate to the chamber and the experimental situation (mean±s.d.): 7.6±1.11 vs. 2.9±0.69 days to feed in the chamber (*shaping*), t-test: t = 3.868, p<0.001. Of the birds that completed autoshaping, there were no significant differences between the groups in the number of days they needed before starting to peck the touch-screen. But 4 of the 11 (36.37%) non-experienced and 7 of the 17 (41.18%) experienced pigeons had to be excluded from experiment because of non-fulfillment of the criteria for successful *autoshaping*: they did not learn to peck the touch-screen. In addition, non-experienced pigeons needed significantly more time (7.6±2.76 days) for completing the *training* (Fig 3), both, in comparison to their *pretraining* (4.0±2.00 days, paired t-test: t = -2.855, p = 0.029) and in comparison to training time of experienced pigeons (3.25±2.375 days, t-test: t = 3.261, p = 0.006).

We did not find significant differences between the groups and/or the different viewing conditions in the *Novel Perspective Tests*. Because of this, and comparable numbers of correct choices as in the regular training, we assumed that both, featural and geometric representations withstood spatial translations and continued with the *tests*.

### Tests

#### (1) Geometry test

**Fig 4:**
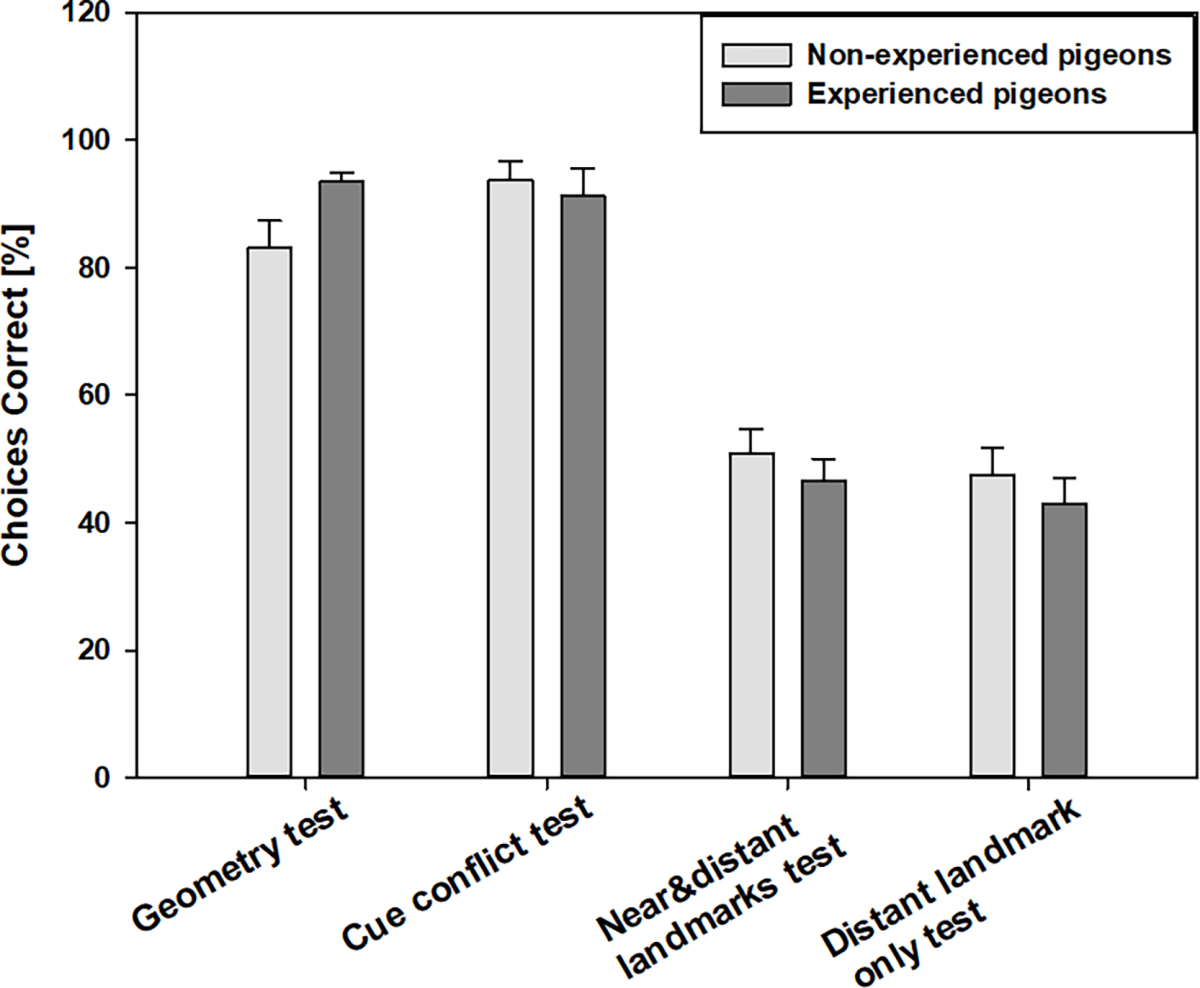
Percentages of choices made to the correct corner (*cue conflict test*: choice of the correct feature/landmark) averaged over all non-experienced (n = 7) and experienced pigeons (n = 10) in the various tests with viewing by both eyes. Error bars represent the standard errors of the mean.

Experienced pigeons did not show significantly more correct choices than non-experienced pigeons (Fig 4, Table 1), (t-test, viewing with both eyes: t = -2.608, p = 0.020; viewing with the left eye: t = -2.373, p = 0.031; viewing with the right eye: t = -2.755, p = 0.015). The number of correct choices in the *geometry test* was always significantly above 50% (the probability of choosing the correct corner by chance), regardless of whether the pigeons used both eyes or just one eye (ANOVA/Fisher-test (LSD), S1 Table).

**Table 1:** Correct choices (%) of experienced and non-experienced pigeons in all tests with all viewing conditions (mean±s.e.m.).

Test	Experienced pigeons (n = 10)	Non-experienced pigeons (n = 7)
*Geometry test*		
Binocular viewing	93.43±2.893	83.03±4.305
Viewing with the left eye	90.62±3.160	75.45±6.229
Viewing with the right eye	92.81±2.797	78.57±4.746
*Cue conflict test*		
Binocular viewing	91.25±4.340	93.75±2.893
Viewing with the left eye	88.13±3.892	91.07±2.499
Viewing with the right eye	87.19±4.356	90.18±2.305
*Near&distant landmarks test*		
Binocular viewing	46.56±3.437	50.89±3.900
Viewing with the left eye	47.19±2.207	42.86±2.334
Viewing with the right eye	42.81±3.549	50.00±2.552
*Distant landmark only test*		
Binocular viewing	42.81±4.168	47.39±4.376
Viewing with the left eye	38.44±2.874	38.02±4.068
Viewing with the right eye	39.06±4.147	44.79±4.320

#### (2) Cue conflict test

There were no significant differences between the two groups of pigeons in the *cue conflict test* (Fig 4, t-test, viewing with both eyes: t = 1.260, p = 0.227; viewing with the left eye: t = 0.574, p = 0.287; viewing with the right eye: t = 0.535, p = 0.601).

**Fig 5:**
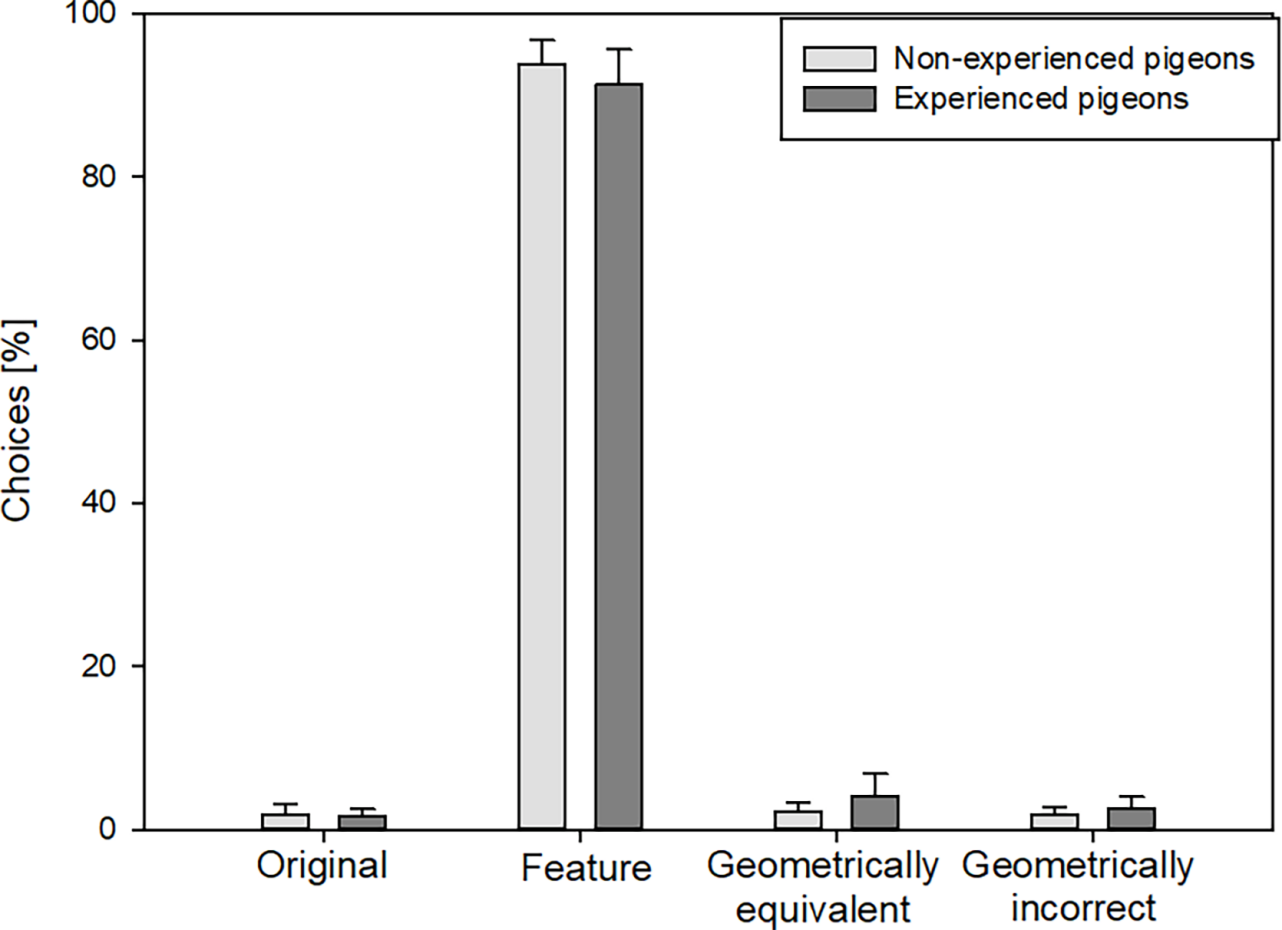
Percentages of choices to the various corners in the *cue conflict test* by non-experienced (n = 7) and experienced pigeons (n = 10) using both eyes. Error bars represent the standard errors of the mean.

The *cue conflict test*, where the features rotated clockwise around the rectangle, showed that both pigeon groups preferred choosing the corner on the basis of landmark information and not on geometrical information (Fig 5). They pecked the corner with the correct feature significantly more often in comparison to the other corners, even though the geometric information at that corner was incorrect (FRM ANOVA on ranks, S2 Table).

The number of choices of the correct feature/landmark in the *cue conflict test* was always significantly above 25% (the probability of choosing the corner by chance), regardless of whether the pigeons used both eyes or just one eye (ANOVA/Fisher-test (LSD), S3 Table).

#### (3) Near & distant landmarks test, (4) distant landmark only test

In the *near & distant landmarks test*, both features with the correct geometric information (the correct feature and the feature of the diagonal corner) were removed. In the *distant landmark only test*, the feature close to the correct corner (“near”, at the end of the short side of the rectangle) was also removed.

In both *landmark tests* there were no significant differences between experienced and non-experienced pigeons (Table 2, t-test, *near & distant landmarks test*: viewing with both eyes: t = 0.825, p = 0.422; viewing with the left eye: t = -1.317, p = 0.204; viewing with the right eye: t = 1.506, p = 0.153; *distant landmark only test*: viewing with both eyes: t = 0.779, p = 0.224; viewing with the left eye: t = -0.086, p = 0.933; viewing with the right eye: t = 0.904, p = 0.381).

**Table 2:** Choices (%) of experienced and non-experienced pigeons in the *near & distant landmarks test* and the *distant landmark only test* (mean±s.e.m.).

*Near&distant* *landmarks test*		correct corner	diagonal corner	near corner	distant corner
Experienced pigeons	Binocular viewing	46.56±3.438	31.25±2.135	12.50±2.234	11.25±3.062
(n = 10)	Left eye	47.19±2.207	31.56±2.734	12.19±1.946	8.44±4.402
	Right eye	42.81±3.549	40.63±3.194	7.19±1.683	7.19±1.322
Non-experienced pigeons	Binocular viewing	50.89±3.900	33.04±3.039	9.82±3.006	8.48±1.767
(n = 7)	Left eye	42.86±2.334	36.61±3.900	12.05±2.499	8.48±6.115
	Right eye	50.00±2.552	32.14±2.950	12.95±4.494	8.48±2.788
*Distant landmark* *only test*		correct corner	diagonal corner	near corner	distant corner
Experienced pigeons	Binocular viewing	42.81±4.168	30.63±2.543	15.31±2.207	10.94±2.519
(n = 10)	Left eye	38.44±2.874	33.13±2.803	17.50±1.693	10.31±2.511
	Right eye	39.06±4.147	31.56±3.681	19.36±2.543	8.75±2.273
Non-experienced Pigeons	Binocular Viewing	47.40±4.376	33.33±3.194	13.02±0.960	6.77±3.168
(n = 7)	Left eye	38.02±4.068	31.77±2.726	22.92±2.234	6.77±1.491
	Right eye	44.79±4.320	28.65±2.604	16.15±3.064	7.81±0.699

In Fig 4 it has already been seen that the performance of both *landmark tests* was poorer in comparison to the other both tests. Both pigeon groups showed significantly more correct choices in the *cue conflict test* and the *geometry test* compared to the *near & distant landmarks test* and the *distant landmark only test* (RM ANOVA: experienced pigeons: F = 57.926, p<0.001; non-experienced pigeons: F = 27.603, p<0.001).

Table 2 also shows that most of the pecks were at the correct corner, followed by the diagonal corner (which had the same correct geometric information) and then the near and the distant corners.

**Fig 6:**
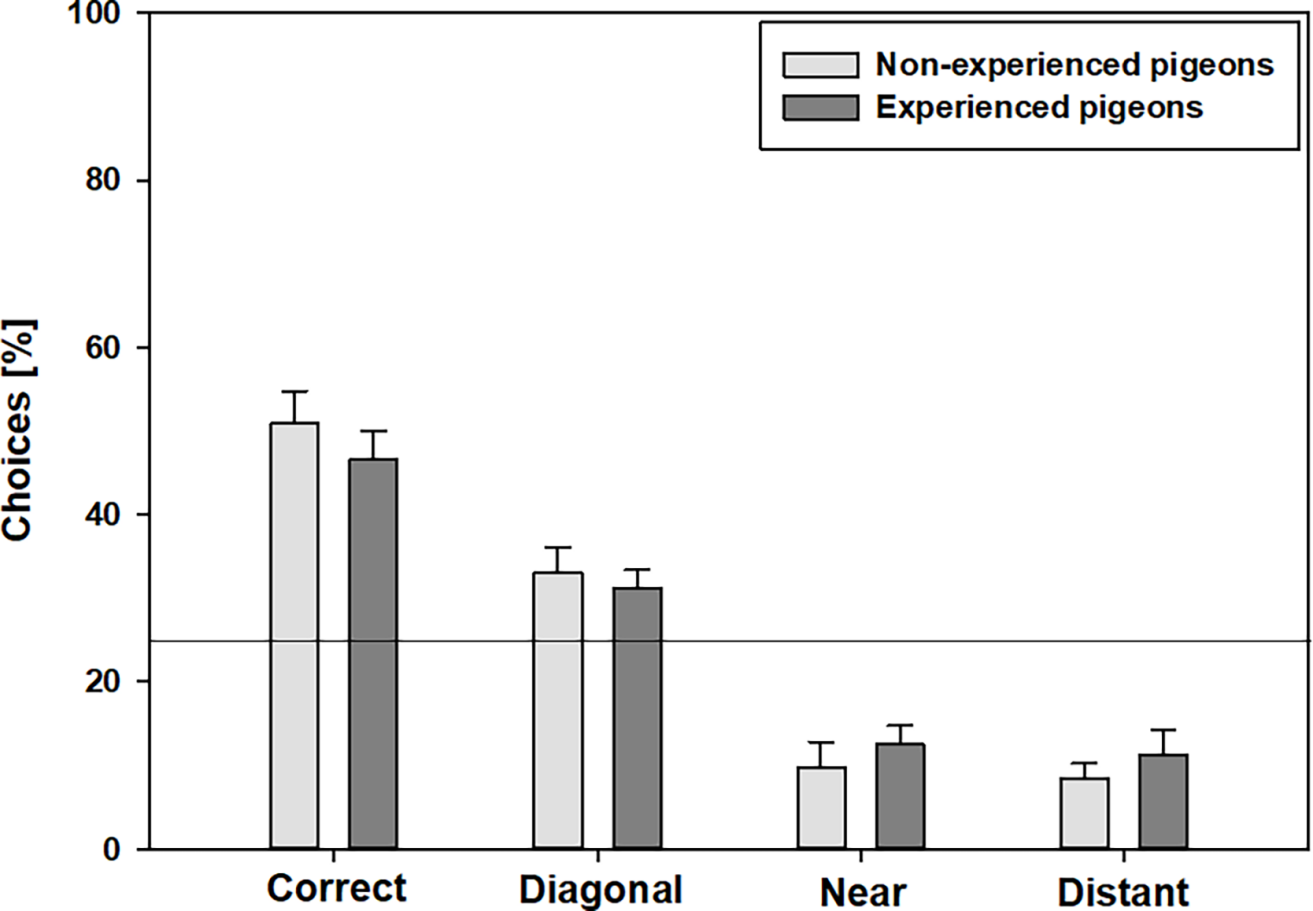
Percentages of choices to the various corners in the *near & distant landmarks test* by non-experienced (n = 7) and experienced pigeons (n = 10) using *both* eyes. Error bars represent the standard errors of the mean. The solid line at 25% indicates the probability of choosing the correct corner by random chance alone.

Generally, both pigeon groups pecked the corner with the correct feature significantly more often in comparison to the other corners (FRM ANOVA on ranks, S4 Table), but the number of pecks to the various corners varied a lot. In detail, neither group differentiated significantly between the correct corner and the diagonal corner and the two corners that were directly neighboring to the correct corner and geometrically incorrect (“near” versus “distant”). This was seen regardless of whether the pigeons used both eyes or just one eye (paired t-test, S5 Table, Fig 6).

The number of correct choices in the *near & distant landmarks test* was always significantly above 25% (the probability of choosing the correct corner by chance) regardless of whether the pigeons used both eyes or only one eye. But in both pigeon groups, the choice of the ***diagonal*** corner did not differ significantly from 25% (random chance), regardless of whether the pigeons used both eyes or just one eye (the number of the choice of the diagonal corner should be significantly under 25%) (S6 Table). Thus, we cannot exclude that the choice of this corner was by chance. Furthermore, the choice of the corner next to the correct one (“near”, at the short end of the rectangle) did not differ significantly from 25% (random chance) if non-experienced pigeons used only their right eye (F = 7.19, p = 0.02).

**Fig 7:**
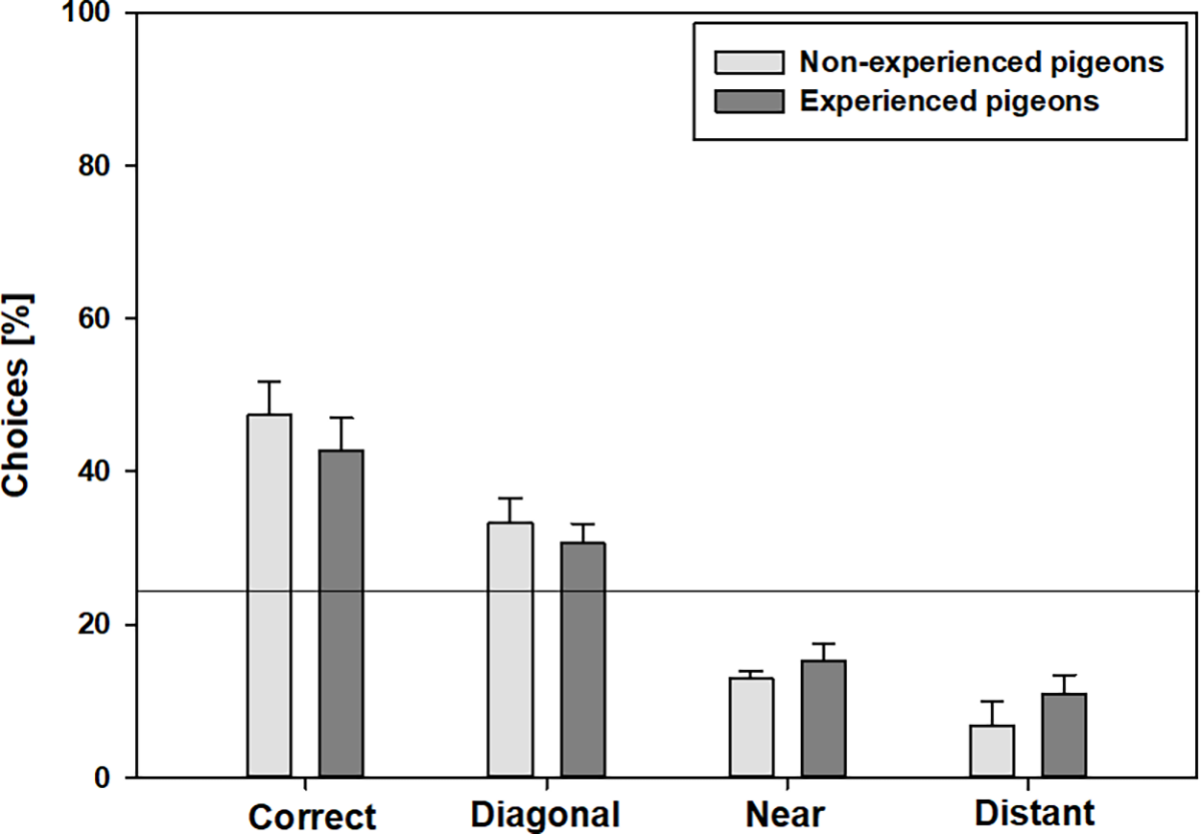
Percentages of choices to the various corners for the *distant landmark only test* by non-experienced (n = 7) and experienced pigeons (n = 10) using *both* eyes. Error bars represent the standard errors of the mean. The solid line at 25% indicates the probability of choosing the correct corner by random chance alone.

In the *distant landmark only test*, only the feature that was adjacent but distant from the correct corner (at the end of the long side of the rectangle) was still shown. Again, both pigeon groups pecked the corner with the correct feature significantly more often in comparison to the other corners (FRM ANOVA on ranks, S7 Table), but the number of pecks to the various corners varied a lot. Like in the *near & distant landmark test* neither group differentiated significantly between the correct corner and the diagonal corner or the corners that were neighboring to the correct corner (“near” and “distant”) regardless of whether the pigeons used both eyes or just one eye (paired t-test, Table 2, S8 Table, Fig 7). Additionally, non-experienced pigeons did not differ significantly between the correct and the near corner when using just one eye (Table 2, S8 Table, paired t-test, Fig 7).

In non-experienced pigeons, the choice of the correct corner was not significantly above 25% (random chance) if they used only their left eye (ANOVA/Fisher-test (LSD), F = 10.25, p = 0.009). So we cannot exclude that the choice of this corner was by chance. Furthermore, like in the *near & distant landmark test* in both pigeon groups, the choice of the diagonal corner was not significantly under 25% (random chance) regardless of whether the pigeons used both eyes or just one eye The choice of the corner next to the correct one (“near”, at the short end of the rectangle) also did not differ significantly from 25% (random chance) if non-experienced pigeons used only their left or their right eye (left eye: F = 0.87, p = 0.373; right eye: F = 8.353, p = 0.016) or if experienced pigeons used only their right eye (F = 4.893, p = 0.04)

## Discussion

The pigeons in this experiment differed in their navigational experience and learned to get food on the basis of information on a touch-screen monitor in an operant chamber. The stimulus was a schematic rectangular environment with landmarks in each corner, which were modified for different tests. Touch-screen experiments differ in several ways from open-field studies, e.g. the search area is relatively small and presented on a vertical display instead of a horizontal surface, and landmarks are not real 3-D objects but 2-D computer drawings, but therefore touch-screen experiments enable us to control variables more rigidly and easily and to conduct a larger number of trials. There are studies which show that at least the encoding of geometric information in chicks, like in children, is specifically based on subtle 3-D cues provided by the terrain instead of by salient 2-D brightness contours on surfaces or columns (chicks: [45]). Also studies with fishes show, that it is not the same whether animals have to orientate in a 2-D environment or in a real 3-D environment with extended surfaces and their properties [46,47]. Besides, the lack of disorientation procedures of the animals in 2D-experiments to avoid the use of inertial information could make comparisons with 3D-experiments difficult. However, there is no reason to underestimate that behaviors seen in an experiment with a touch-screen reflect general capabilities that would also be seen in the natural environment [48].

We found that non-experienced pigeons needed more time to habituate to the experimental situation and failed to do so more often. This could be due to their limited contact with novel situations and a higher stress level. Whereas experienced pigeons needed a similar number of days for pretraining and training, non-experienced pigeons needed significantly more days for training. It seems that experienced pigeons, which were familiar with combining a variety of different kinds of information during their flights, conjoin the relation between the response key and feature information already during pretraining. Perhaps in non-experienced pigeons this conjunction did not happen until the training phase. This might be an indication that experience (of any type) or the extent of exposure to environmental cues positively influences cognitive flexibility.

The *geometry test* (and also the *landmark tests*) showed that both pigeon groups were able to orientate on the basis of landmark and geometric information. In addition, the pigeons were able to conjoin landmark and geometric information, which is consistent with the results of Kelly et al. [4] and Kelly & Spetch [19]. They found e.g. that pigeons trained with features showed systematic rotational errors when the features were removed, indicating that they had indeed encoded geometry, even though this was not required to solve the task. We expected that navigational experience has an influence on encoding geometric information, but this was not verified in the frame of the present study. This is in contrast with findings in fishes, which showed that rearing conditions or rearing environment can alter the preferential use of geometric and landmark information [49] but is in line with findings in chicks. Several studies suggest, that the ability to deal with geometry seems to depend more on predisposed mechanisms than on rearing conditions and experience after hatching [11,50,51,52]. In chicks, the hippocampal formation seems to be recruited to support geometrical computations needed for goal orientation in a rectangular environment [53] and in another laboratory study, hippocampus-lesioned pigeons were insensitive to geometric information and relied exclusively on landmark information [26]. Following this, and the fact that pigeons with navigational experience have a larger hippocampus than pigeons without navigational experience [24], we expected a more distinct difference in encoding geometric information between the two groups. But it seems to be that, at least in a 2-D environment, the generally enlarged hippocampus of homing pigeons in contrast to other pigeon breeds and/or bird species [54] is sufficient for encoding geometric information.

The absence of hemispheric asymmetry (there were no differences between left-eye viewing and right-eye viewing) during the *geometry test* indicates that geometric information is encoded in both hemispheres. This is consistent with laboratory studies with homing pigeons [55] that showed that both hemispheres might be involved in encoding of geometric information, with the left hemisphere encoding absolute distance and the right hemisphere encoding relational geometry.

The *cue conflict test* placed geometric and landmark information in conflict, rather than removing the landmarks entirely. The *cue conflict test* showed that both pigeon groups preferred using landmark information, even if it was contradicted by correct geometric information. This is consistent with previous studies in chicks and fish [10,12,56] and one study in pigeons [55] but contrary to two others in pigeons [4,19]. Using a similar experimental design as we did, the study of Kelly & Spetch [19] showed that when geometry and landmarks provided conflicting information, pigeons divided their choices equally between the location defined by the correct landmark and the location defined by the correct geometry. One possibility for this discrepancy between their research and ours might be the breed of used birds: homing pigeons in our study versus Silver Kings in the Kelly et al. studies. Homing pigeons are selectively bred for accurate homing behavior, whereas, Silver Kings are typically bred for laboratory use or meat production and too large to fly long distances. Thus, it is not unlikely, that they not just differ in their homing behavior, but also in encoding geometric and featural information.

In the present study, landmark information overshadowed the geometric information even in the pigeons that had spent their whole life in the loft. Overshadowing (defined as the predominance of one kind of information over other kinds) is widely observed in the spatial domain, including in pigeons [57]. Our results indicate that this effect is not dependent on individual life history, at least in homing pigeons. Furthermore, neither group showed hemispheric asymmetry, which means that both hemispheres weight landmarks more relevant than geometry.

The *landmark tests* investigated whether the pigeons also use landmarks other than the landmark in the correct corner for orientation. Both pigeon groups demonstrated in principle, the ability to also use distant features to correctly locate the rewarded corner and chose the correct corner significantly more often than the other corners, but performance was much worse in comparison to the other tests. This is consistent with a previous study on pigeons [19] but contrasts with studies on chicks [10] and rats [7] which showed that these species have failed to show learning of the features in the geometrically incorrect corners. A plausible explanation may be the inhabitation of contrasting ecological niches. The lack of differences between experienced and non-experienced pigeons indicates that encoding landmark information is not dependent on experience of navigating long distance over extended landscapes. Interestingly, the differentiation between the various corners was difficult. The number of choices between the correct and the diagonal and the near and the distant feature did not differ significantly and in the *distant landmark only test* non-experienced pigeons even had problems with the differentiation of the correct corner and near corner if they used just the left or right eye. It looks very much that either the pigeons could not use landmark and geometry information together, or that the conjunction of several features is important for orientation, with each landmark being a crucial element, the absence of which will result in poorer orientation performance. The second interpretation also explain the fact that the choice of the different corners often does not significantly exceed the 25% level of random chance. This is also consistent with the results of the *cue conflict test*, which demonstrated the pigeons’ preference for landmarks. Non-experienced pigeons had more problems with the differentiation of the various corners and this might be an indication that experience or the extent of exposure to environmental cues positively influences the capability to conjunct several features for orientation.

We found hemispheric asymmetries particularly in non-experienced pigeons in both *landmark tests*. In non-experienced pigeons the differentiation between the various corners was worse under monocular occlusion. This was already seen in the comparison with the probability of choosing the correct corner by chance (25% or 50%). In the *distant landmark only test* this was even more distinct, but without a clear superiority for one eye respectively one brain hemisphere. It seems that the viewing condition became more important for non-experienced pigeons when less featural information (especially features) was available. Or, vice versa, the degree of experience became more important when less featural information was available. Many studies about avian brain lateralization are based on chicks, and it is known that there are differences between chick and pigeons not only in regards to brain morphology in general but also specifically for lateralization [23,56,58]. This might not be too surprising, since chickens and pigeons develop differently (altricial vs. precocial) and also show profound differences in the use of space under natural conditions (ground living vs. flying over long distances). It has been suggested that chicks process spatial information primarily in the right hemisphere [59,60]. In contrast, several studies on pigeons have shown a left-hemispheric superiority for navigation and/or discrimination of various visual objects [15,41,61]. Thus, it should be expected that test performance would be better with the right eye than with the left, but our study did not support that. Generally, we cannot constitute a clear hemispheric superiority, but there were differences between the differentiation of the various corners in both landmark tests using both eyes or just one eye. The organization of processing spatial information in the left or right hemisphere seems to be more diverse and it is difficult to generalize which kind of information will be processed in which hemisphere. That diversity is supported here by the different results of the both *landmark tests* under monocular occlusion.

That there is a high diversity in the organization of processing spatial information in the different hemispheres was also the assumption of Wilzeck et al. [54]. On the one hand, the authors also showed a pattern for the hemispheric encoding of geometric and landmark information by the pigeon that was different than the pattern previously reported for the domestic chick. On the other hand, they showed that the relative use of geometry and landmarks depended on experience. However, this referred to different experiences acquired by the pigeons during different conditions in a kind of pretraining (where there was just geometric information available or geometric and landmark information available) and thus was not directly comparable with the kind of experience of the pigeons in the present study. But the authors found an experience-dependent effect, and that is consistent with the role of experience in the present study.

Generally, results with chicks have shown clear differences in spatial processing (encoding relational spatial information and absolute metric information) between left-hemispheric and right-hemispheric use as well as little differences between right-hemispheric and binocular use [62]. Yet pigeons in the study of Wilzeck et al. [54] showed virtually no difference between left-hemispheric and right-hemispheric use (and no distinct left-hemispheric dominance) but did show a difference between the binocular condition and each of the monocular conditions [54]. That was just in part shown in both *landmark tests* of the present study. Differences between binocular and monocular viewing were observed only in the *landmark tests* and here particularly in non-experienced pigeons. Thus, the *landmark tests* showed that the condition of binocular or monocular viewing could have, after all, an influence on encoding geometric or landmark information. Those tests also showed that encoding is influenced by experience, because the choice of the four various corners differed between experienced and non-experienced pigeons and the different viewing conditions, but without a clear tendency to one hemisphere. A stronger representation of visual stimuli in the pigeons’ left hemisphere, which was reported in earlier studies in various laboratory settings [38,41,61,63], could not be confirmed. We expected that at least experienced pigeons would be able to differentiate better between the four various corners if they could use their right eye and thus, their left hemisphere. But that was not the case. Perhaps more realistic experiments in a 3-D environment, or even free-flight experiments, could provide more insight about this phenomenon. Also repeated discrimination tasks might be able to help clarify this, because Verhaal et al. [64] has shown that differential left-right performance is already present before learning but increases substantially as soon as some cues are associated with a reward. The results of the present study support the conclusion that each hemisphere of the pigeon brain encodes multiple cues that may allow the bird to process orientation tasks in a fast and flexible manner.

In conclusion we can say that, in a 2-D-environment, pigeons are able to encode geometric and landmark information for orientation but prefer using landmark information. In the present study, there is no influence of navigational experience on encoding geometric and landmark information. Encoding geometric or featural information cannot be assigned to the left or right hemisphere. Either it is processed in both brain hemispheres, or, in case of processing geometric information in one hemisphere and landmark information in the other, both processes are integrated before response. More investigations are necessary to clarify these open questions. The conjunction of geometric and landmark information is important for orientation, probably processed in both hemispheres and influenced by navigational experience.

## Supporting information

S1 Table Statistical results of comparisons between the choices of the correct corners and the probability of choosing the corner by chance (50%) in the *geometry test* (ANOVA/Fisher’s least significance difference test (LSD)). (DOCX)

S2 Table Statistical results of comparisons between the choice of the correct feature and the other corners (FRM ANOVA on ranks) in the *cue conflict test*. (DOCX)

S3 Table Statistical results of comparisons between the choices of the correct landmark and the probability of choosing the corner by chance (25%) in the *cue conflict test* (ANOVA/ Fisher’s least significance difference test (LSD)). (DOCX)

S4 Table Statistical results of comparisons between the choice of the correct feature and the other corners (FRM ANOVA on ranks) in the *near & distant landmarks test*. (DOCX)

S5 Table Statistical results of comparisons between the choices of the various corners in the *near & distant landmarks test* (paired t-test). (DOCX)

S6 Table Statistical results of comparisons between the choices of the *diagonal* corner and the probability of choosing the corner by chance (25%) in the *landmarks test near & distant* and the *distant landmark only test* (ANOVA/ Fisher’s least significance difference test (LSD)). (DOCX)

S7 Table Statistical results of comparisons between the choice of the correct feature and the other corners (FRM ANOVA on ranks) in the *distant landmark only test*. (DOCX)

S8 Table Statistical results of comparisons between the choices of the various corners in the *distant landmark only test* (paired t-test). (DOCX)
