# Supplementary material for: The orientation of homing pigeons (Columba livia f.d.) with and without navigational experience in a two-dimensional environment
Source: PLoS One. 2017 Nov 27;12(11):e0188483. doi: 10.1371/journal.pone.0188483 (PMC5703563; doi:10.1371/journal.pone.0188483)
Supplement: S1 Table — (DOCX) [file pone.0188483.s001.docx]

**S1 Table. Statistical results of comparisons between the choices of the correct corners and the probability of choosing the corner by chance (50%) in the *geometry test* (ANOVA/ Fisher’s least significance difference test (LSD)).**

| ***Geometry Test*** | **Experienced pigeons**  **(n=10)** | **Non-experienced pigeons (n=7)** |
| --- | --- | --- |
| *Geometry test* |  |  |
| Binocular viewing | F=832.00, p<0.001 | F=58.88, p<0.001 |
| Viewing with the left eye | F=165.32, p<0.001 | F=16.69, p=0.002 |
| Viewing with the right eye | F=234.28, p<0.001 | F=36.25, p<0.001 |
