# Supplementary material for: The orientation of homing pigeons (Columba livia f.d.) with and without navigational experience in a two-dimensional environment
Source: PLoS One. 2017 Nov 27;12(11):e0188483. doi: 10.1371/journal.pone.0188483 (PMC5703563; doi:10.1371/journal.pone.0188483)
Supplement: S3 Table — (DOCX) [file pone.0188483.s003.docx]

**S3 Table. Statistical results of comparisons between the choices of the correct landmark and the probability of choosing the corner by chance (25%) in the *cue conflict test* (ANOVA/ Fisher’s least significance difference test (LSD)).**

| **Test** | **Experienced pigeons**  **(n=10)** | **Non-experienced pigeons (n=7)** |
| --- | --- | --- |
| *Cue conflict test* |  |  |
| Binocular viewing | F=233,00, p<0.001 | F=564.67, p<0.001 |
| Viewing with the left eye | F=263.06, p<0.001 | F=699.06, p<0.001 |
| Viewing with the right eye | F=203.78, p<0.001 | F=799.35, p<0.001 |
