# Supplementary material for: The orientation of homing pigeons (Columba livia f.d.) with and without navigational experience in a two-dimensional environment
Source: PLoS One. 2017 Nov 27;12(11):e0188483. doi: 10.1371/journal.pone.0188483 (PMC5703563; doi:10.1371/journal.pone.0188483)
Supplement: S4 Table — (DOCX) [file pone.0188483.s004.docx]

**S4 Table. Statistical results of comparisons between the choice of the correct feature and the other corners (FRM ANOVA on ranks) in the *near & distant landmarks test*.**

| **Test** | **Experienced pigeons**  **(n=10)** | **Non-experienced pigeons (n=7)** |
| --- | --- | --- |
| *Cue Conflict test* |  |  |
| Binocular viewing | Chi-square=25.125, p>0.001 | Chi-square=18.313, p>0.001 |
| Viewing with the left eye | Chi-square=26.455, p>0.001 | Chi-square=16.886, p>0.001 |
| Viewing with the right eye | Chi-square=24.612, p>0.001 | Chi-square=18.838, p>0.001 |
