# Supplementary material for: The orientation of homing pigeons (Columba livia f.d.) with and without navigational experience in a two-dimensional environment
Source: PLoS One. 2017 Nov 27;12(11):e0188483. doi: 10.1371/journal.pone.0188483 (PMC5703563; doi:10.1371/journal.pone.0188483)
Supplement: S5 Table — (DOCX) [file pone.0188483.s005.docx]

**S5 Table. Statistical results of comparisons between the choices of the various corners in the *near & distant landmarks test* (paired t-test)*.***

| **Experienced pigeons (n=10)**  > using **both** eyes | diagonal | near | distant |
| --- | --- | --- | --- |
| correct | t=3.495, p=0.007 | t=6.452, p<0.001 | t=6.188, p<0.001 |
| diagonal |  | t=5.636, p<0.001 | t=4.520, p<0.001 |
| near |  |  | t=0.440, p=0.670 |
| > using **left** eye |  |  |  |
| correct | t=3.822, p=0.004 | t=10.058, p<0.001 | t=13.458, p<0.001 |
| diagonal |  | t=4.751, p=0.001 | t=6.101, p<0.001 |
| near |  |  | t=1.724, p=0.119 |
| > using **right** eye |  |  |  |
| correct | t=0.376, p=0.716 | t=7.528, p<0.001 | t=8.819, p<0.001 |
| diagonal |  | t=8.638, p<0.001 | t=8.341, p<0.001 |
| near |  |  | t=0.000, p=1.000 |
| **Non-experienced pigeons (n=7)**  > using **both** eyes | diagonal | near | distant |
| correct | t=3.230, p=0.018 | t=6.634, p<0.001 | t=8.554, p<0.001 |
| diagonal |  | t=4.400, p=0.005 | t=6.049, p<0.001 |
| near |  |  | t=0.528, p=0.617 |
| > using **left** eye |  |  |  |
| correct | t=1.247, p=0.259 | t=9.758, p<0.001 | t=7.432, p<0.001 |
| diagonal |  | t=3.990, p=0.007 | t=4.930, p=0.003 |
| near |  |  | t=1.039, p=0.339 |
| > using **right** eye |  |  |  |
| correct | t=4.155, p=0.006 | t=6.256, p<0.001 | t=8.549, p<0.001 |
| diagonal |  | t=3.403, p=0.014 | t=4.964, p=0.003 |
| near |  |  | t=0.795, p=0.457 |
