# Supplementary material for: The orientation of homing pigeons (Columba livia f.d.) with and without navigational experience in a two-dimensional environment
Source: PLoS One. 2017 Nov 27;12(11):e0188483. doi: 10.1371/journal.pone.0188483 (PMC5703563; doi:10.1371/journal.pone.0188483)
Supplement: S6 Table — (DOCX) [file pone.0188483.s006.docx]

**S6 Table. Statistical results of comparisons between the choices of the *diagonal* corner and the probability of choosing the corner by chance (25%) in the *landmarks test near & distant* and the *distant landmark only test* (ANOVA/ Fisher’s least significance difference test (LSD)).**

| **Test** | **Experienced pigeons**  **(n=10)** | **Non-experienced pigeons (n=7)** |
| --- | --- | --- |
| *Landmarks test near&distant* |  |  |
| Binocular viewing | F=8.57, p=0.009 | F=6.99, p=0.021 |
| Viewing with the left eye | F=5.76, p=0.027 | F=8.86, p=0.012 |
| Viewing with the right eye | F=23.94, p<0.001 | F=5.86, p=0.032 |
| *Distant landmark only test* |  |  |
| Binocular viewing | F=4.89, p=0.040 | F=6.81, p=0.026 |
| Viewing with the left eye | F=8.40, p=0.010 | F=6.17, p=0.032 |
| Viewing with the right eye | F=3.18, p=0.092 | F=1.96, p=0.192 |
