# Supplementary material for: The orientation of homing pigeons (Columba livia f.d.) with and without navigational experience in a two-dimensional environment
Source: PLoS One. 2017 Nov 27;12(11):e0188483. doi: 10.1371/journal.pone.0188483 (PMC5703563; doi:10.1371/journal.pone.0188483)
Supplement: S8 Table — (DOCX) [file pone.0188483.s008.docx]

**S8 Table. Statistical results of comparisons between the choices of the various corners in the *distant landmark only test* (paired t-test)*.***

| **Experienced pigeons (n=10)**  > using **both** eyes | diagonal | near | distant |
| --- | --- | --- | --- |
| correct | t=2.028, p=0.073 | t=4.627, p=0.001 | t=5.517, p<0.001 |
| diagonal |  | t=5.233, p<0.001 | t=4.815, p<0.001 |
| near |  |  | t=1.382, p=0.200 |
| > using **left** eye |  |  |  |
| correct | t=1.118, p=0.293 | t=5.154, p<0.001 | t=6.364, p<0.001 |
| diagonal |  | t=4.564, p=0.001 | t=4.824, p<0.001 |
| near |  |  | t=2.501, p=0.034 |
| > using **right** eye |  |  |  |
| correct | t=1.026, p=0.332 | t=3.533, p=0.006 | t=5.796, p<0.001 |
| diagonal |  | t=2.736, p=0.023 | t=4.778, p=0.001 |
| near |  |  | t=3.188, p=0.011 |
| **Non-experienced pigeons (n=7=**  > using **both** eyes | diagonal | near | distant |
| correct | t=2.058, p=0.095 | t=7.911, p<0.001 | t=5.738, p=0.002 |
| diagonal |  | t=5.278, p=0.003 | t=5.937, p=0.002 |
| near |  |  | t=1.936, p=0.111 |
| > using **left** eye |  |  |  |
| correct | t=1.035, p=0.348 | t=2.586, p=0.049 | t=6.202, p=0.002 |
| diagonal |  | t=2.371, p=0.064 | t=7.108, p<0.001 |
| near |  |  | t=10.826, p<0.001 |
| > using **right** eye |  |  |  |
| correct | t=2.847, p=0.036 | t=4.088, p=0.009 | t=8.753, p<0.001 |
| diagonal |  | t=2.981, p=0.031 | t=8.771, p<0.001 |
| near |  |  | t=2.390, p=0.062 |
